# An Unusual Presentation of Jejunal Diverticulitis Mimicking Acute Cholecystitis

**DOI:** 10.7759/cureus.80682

**Published:** 2025-03-16

**Authors:** Henry M Olivera-Perez, Anna Huang Perez, Andrea Gochi, Spencer Tagg, Emily Miraflor

**Affiliations:** 1 Department of Surgery, University of California, San Francisco - East Bay, Oakland, USA

**Keywords:** complicated acute cholecystitis, jejunal diverticulitis, jejunal diverticulosis, severe thrombocytopenia, small bowel diverticulosis

## Abstract

Jejunal diverticulosis is a rare entity and jejunal diverticulitis is even rarer. Here we review a patient who presented with right upper quadrant pain and elevated white blood cell count, mimicking acute cholecystitis, as well as thrombocytopenia (21x10^3/mcl). The patient was managed non-operatively with antibiotics and had improvement in symptoms and thrombocytopenia. Our study highlights the importance of maintaining jejunal diverticulitis in the differential for right upper quadrant pain, and its association with severe thrombocytopenia.

## Introduction

Diverticulosis of the large bowel is a commonly encountered entity. Jejunal diverticulosis, however, is an uncommon pathology that is often asymptomatic. Although there is no established imaging gold standard, computed tomography (CT) is the most commonly used modality in the acute state [1]. Given its rarity, it is often not maintained on the differential when evaluating abdominal pain [2], despite the fact that it can have devastating complications including perforation [3]. In cases where jejunal diverticulitis does cause abdominal pain, it typically manifests as peri-umbilical or vague abdominal pain [4-6]. One study reported an association between jejunal diverticulitis and thrombocytopenia [7]. Here, we report a case of a patient who presented with right upper quadrant pain, nausea/vomiting, leukocytosis, and severe thrombocytopenia and was found to have acute jejunal diverticulitis.

## Case presentation

A 74-year-old male with a history of diffuse large B-cell lymphoma in remission, hypertension (HTN), and left inguinal hernia repair (one year prior to presentation) presented to the emergency department with abdominal pain.

**Figure 1:**
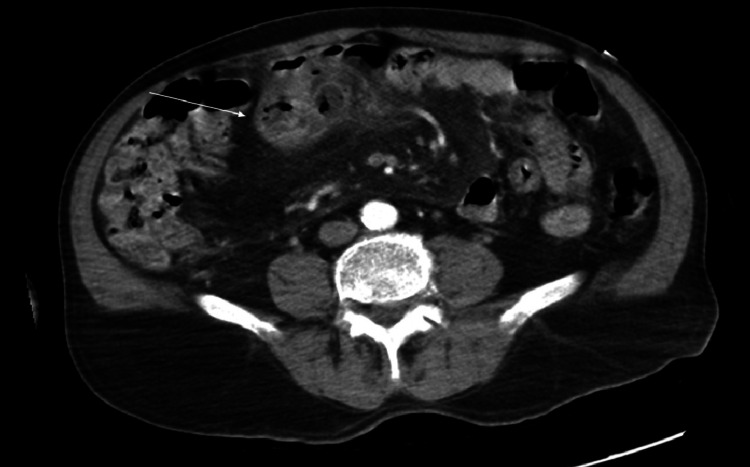
Computed tomography (CT) demonstrating acute jejunal diverticulitis (white arrow).

The patient presented to the emergency department (ED) after experiencing left-sided abdominal pain for two weeks, which resolved for one week before the development of new epigastric and right upper quadrant pain, accompanied by nausea and vomiting. On exam, the patient was tender to the epigastric region and right upper quadrant. He was found to have leukocytosis to 16 x10^3/mcl and a platelet count of 21 x10^3/mcl (patient baseline 200 x10^3/mcl). The platelet count was confirmed by the laboratory via peripheral smear and microscopy. A computed tomography (CT) of the abdomen and pelvis with IV contrast was obtained showing inflammatory fat stranding around a jejunal diverticula in the right upper quadrant consistent with acute jejunal diverticulitis (Figure 1), without abscess noted. Duodenal and colonic diverticulosis was noted without diverticulitis. There was a concern for gallbladder wall thickening on CT, therefore a right upper quadrant ultrasound was ordered showing normal gallbladder wall thickness and cholelithiasis without cholecystitis. Magnetic resonance cholangiopancreatography (MRCP) was performed to further evaluate for cholecystitis; the MRCP showed cholelithiasis with no evidence of cholecystitis. The patient was started on intravenous ceftriaxone and metronidazole for six days and discharged on oral amoxicillin/clavulanate for a total 10-day course of antibiotics. His abdominal pain improved during his hospitalization. His platelet count was trended daily and had improved to 263 x10^3/mcl by hospital Day 6 while on IV antibiotics and without any need for transfusion. The patient was discharged with outpatient follow-up. One year after hospitalization, the patient did not experience a recurrence of symptoms.

## Discussion

Jejunal diverticulitis is an uncommon entity compared to colonic diverticulitis, with older men as the primary affected population, similar to our patient. Prevalence of small bowel diverticulosis has been noted to be up to 1.9% during autopsy [7]. It can present as a life-threatening abdominal infection or as uncomplicated abdominal pain. Currently, there are no established guidelines for the treatment of jejunal diverticulitis [8-10]. Studies have shown a variety of antibiotic regimens for treating jejunal diverticulitis, with our patient demonstrating clinical improvement after a six-day course of IV antibiotics [11]. Here we review a patient case of jejunal diverticulitis that presented as classic acute cholecystitis with right upper quadrant pain, nausea, vomiting, elevated white blood cell count (WBC), and thrombocytopenia, highlighting the importance of maintaining jejunal diverticulitis in the differential for right upper quadrant pain. 

In the case of severe thrombocytopenia, there are many different causes and etiologies that could contribute to its development. It can be a sign of intra-abdominal infection and perforation [12], with some theories suggesting that bacteria and sepsis can induce consumptive thrombocytopenia [13-14]. Thrombocytopenia has been reported in approximately 8% of patients with intra-abdominal infections [15], with the risk of mortality increasing with increasing severity of thrombocytopenia [16]. Our patient did not have evidence of perforation on imaging or exam and was able to be managed medically during his hospitalization. This highlights the varied presentations of jejunal diverticulitis, its association with thrombocytopenia, and the importance of further investigation into this uncommon disease.

## Conclusions

Uncommon etiologies of abdominal pain and thrombocytopenia such as jejunal diverticulitis should be considered in the differential diagnosis when evaluating right upper quadrant pain. Severity can range from medically managed abdominal pain to perforation, necessitating emergency surgery with bowel resection. Further studies on jejunal diverticulitis are needed to further characterize and manage this rare disease.
